# Design and Construction of a Double Inversion Recombination Switch for Heritable Sequential Genetic Memory

**DOI:** 10.1371/journal.pone.0002815

**Published:** 2008-07-30

**Authors:** Timothy S. Ham, Sung K. Lee, Jay D. Keasling, Adam P. Arkin

**Affiliations:** 1 Department of Bioengineering, University of California, Berkeley, California, United States of America; 2 Physical Biosciences Division, Lawrence Berkeley National Laboratory, Berkeley, California, United States of America; 3 Department of Chemical Engineering, University of California, Berkeley, California, United States of America; University of British Columbia, Canada

## Abstract

**Background:**

Inversion recombination elements present unique opportunities for computing and information encoding in biological systems. They provide distinct binary states that are encoded into the DNA sequence itself, allowing us to overcome limitations posed by other biological memory or logic gate systems. Further, it is in theory possible to create complex sequential logics by careful positioning of recombinase recognition sites in the sequence.

**Methodology/Principal Findings:**

In this work, we describe the design and synthesis of an inversion switch using the fim and hin inversion recombination systems to create a heritable sequential memory switch. We have integrated the two inversion systems in an overlapping manner, creating a switch that can have multiple states. The switch is capable of transitioning from state to state in a manner analogous to a finite state machine, while encoding the state information into DNA. This switch does not require protein expression to maintain its state, and “remembers” its state even upon cell death. We were able to demonstrate transition into three out of the five possible states showing the feasibility of such a switch.

**Conclusions/Significance:**

We demonstrate that a heritable memory system that encodes its state into DNA is possible, and that inversion recombination system could be a starting point for more complex memory circuits. Although the circuit did not fully behave as expected, we showed that a multi-state, temporal memory is achievable.

## Introduction

Synthetic Biology aims to make the implantation of new, complex biological function in cells more of an engineering science than a biology research project. A central effort of this emerging field is the design of modular biological parts that facilitate both the ease of manufacture and the design of predictable function in cells. The hope is that this will allow applications at larger scale and with more sophistication than is currently feasible with the more ad hoc genetic engineering approaches commonly applied today. If successful, a great deal is to be gained from this approach in supporting classical applications such as industrial expression of useful proteins, creation of pathways for biological synthesis of organic molecules for pharmaceuticals and commodity natural products, or to confer a simple phenotype such as pest or drought resistance on a host.

The field, however, looks forward to challenges in global health, energy and the environment that could be well-served by carefully engineered microbes with more complex “programming” to sense and respond to variable and uncertain environments found beyond the bioreactor. Such applications might include, for example, controlled, safety-assured deployment of engineered microorganisms to remediate water and soils, support crop growth in marginal soil, treat disease, or even serve as cheap emergency blood substitutes as one recent undergraduate iGEM team imagined [1].

These applications will require more sophisticated sensing, actuating and regulatory logic than chemical production pathways and may depend on the temporal history of events experienced by the cell. That is, it may be necessary to “remember” past inputs rather than simply compute and actuate on the present ones. This biological circuitry would be more akin to a sequential logical system, a prerequisite for complex computation, than a combinational one such as simple Boolean logic. Regulatory networks with such memory are relatively common in those natural systems that control, for example, development. Memory of past environments can also confer a fitness advantage in some situations[2], and synthetic biological two-state memory switches have been built in both prokaryotes[3]and eukaryotes [4]. It is clear from the complexity of both natural cellular networks and the oncoming synthetic biological applications that regulatory circuitry of some sophistication is required to sense and survive in the outside world.

Systems whose output depends not only on the current inputs but the input history are necessary for sophisticated computation and information storage. While it is unlikely we will use bacteria as super-computers for a number of reasons, it is interesting to note that the chemical networks that underlie their behavior are formally capable of Turing-machine like computations [5], [6], [7], [8]. In these systems, the inputs are generally chemical “inducers”, the machine is a set of chemical reactions, and the state is generally the stationary-state concentrations of the internal chemical species. For synthetic biological applications in the foreseeable future, we will not likely need to design networks with extremely large computational power. But scaling to larger applications, with more states and deeper sequential logics is certainly a future need.

In synthetic biological applications to date, the logic of the designed regulatory systems is generally simple and implemented by a handful of genetic “parts” [9]. In most cases, these elements are the standard set of less than five work-horse promoters such as the tetracycline and lactose inducible promoters and their cognate transcription factors. These promoters have been studied and even modified slightly for years rendering them useful for gene expression control. There is an ever increasing bestiary of naturally occurring bacterial promoters and some efforts to engineer synthetic promoters and transcription factors for more complex logical functions. However, the heterogeneity in behavior of these devices and undeveloped rules for their composition make designing larger networks with these components unpredictable. In almost all applications there are unreasonable number of cycles of design and testing before the system works as desired.

Design of parts families for which it is relatively simple to construct a new functionally-independent member from knowledge of the current members has become a key focus of foundational synthetic biology. Examples include the rational design of RNA-based mRNA translation regulation and metabolic sensing [10], [11], modular systems for controlling mRNA stability [12], and a designable way of implementing RNAi-based combinations circuits in mammalian cells [13]. While there are still subtleties and a variety of case-by-case issues, these all exploit the relatively simple structural rules in RNA, Watson-Crick complimentarity rules, and the relative ease of RNA *in vitro* evolution to design families of parts with more or less predictable function. There has also been progress in engineering protein-based parts families. It is becoming ever more possible to, for example, design zinc finger proteins as transcription factors [14] or to gain control of eukaryotic scaffolding proteins to design signaling switches [15] and more.

Even with the increasing sophistication of synthetic biological parts families, challenges remain in the scalable design of complex regulatory circuitry. Because of the lack of spatial addressing of “signals” between components, like wires between physically separated components in an electronic circuit, it is generally not possible to reuse the same biological component in the same way as one could reuse a transistor (exceptions include such things as ribosome binding sites to some degree [16]). This leads to heterogeneity of “device physics” across the circuit, as every part is somewhat different than every other and a necessity to actually *have* a chemically different part for every elementary operation of the circuit. The properties of these parts are often very complex, thereby making abstraction into useful mathematics for design, such as Boolean logics, difficult. Further, implementing all these different parts in a cell, were they available, might require fairly large outlays of DNA real estate and place large energetic loads on the cell. Finally, in most current synthetic biological applications, the “states” of the regulatory circuitry are encoded in transient chemical concentrations that require energy outlay to hold and can't be maintained after cell death or transmitted easily from one cell to another.

For all these reasons above, circuits that might operate by changing DNA sequence using, for example, recombinases are attractive complements to gene expression and protein interaction networks. Operations on DNA tend to change state (sequence) in a discrete, almost Boolean, fashion. The state may be maintained without constant energetic input, persists after cell death and even if rare in a cell population a particular sequence may be amplified out from the background by PCR. Further, since the “state” is encoded in DNA, it is possible by the various mechanisms of inter-cell DNA transfer, such as conjugation to pass state-output among cells as a complex form of communication as one iGEM team recently suggested [17]. More subtly, as shown below, the ability to decide the spatial arrangement of recombination sites provides the ability to create circuits with large sequential state spaces accessible from relatively few recombinase inputs with efficient use of DNA.

Motivated by these advantages, in this work, we have explored the use of bacterial inversion recombination systems to construct genetic switches with the above properties and show how more complicated finite-state machine-like devices could be constructed out of such systems. For our purposes invertases are a nice starting point since they act very much as simple switches that may invert a sequence of DNA in place. To summarize briefly, inversion recombination happens between two short inverted repeated DNA sequences, typically less than 30 basepairs (bp) long. The recombinases bind to these inverted repeated sequences, which are recombinase specific. A DNA loop formation, assisted by DNA bending proteins, brings the two repeat sites together, at which point DNA cleavage and ligation occur. This reaction is ATP independent, but requires super-coiled DNA. The end result of the recombination is that the stretch of DNA between the repeated sites inverts. That is, the stretch of DNA switches orientation—what was the coding strand is now the non-coding strand, and vice versa. In this reaction, the DNA is conserved, and no gain or loss of DNA occurs. Additionally, there is great flexibility in the distance between the inverted repeats, ranging from only a few hundred up to 5 kilobases (kb) of additional DNA between the repeats possible (refer to the work of Johnson [18] and Blomfield [19] for a detailed treatment of inversion systems). The advantages of site-specific inversion are the binary dynamics, the sensitivity of output, the efficiency of DNA usage, and its persistent DNA encoding, even after cell death. The disadvantages are the possible interference between multiple recombinases, DNA loss by excision, and reversibility of the reaction.

Previously, we have described a tightly regulated expression switch using the FimE protein of the *fim* system of *E. coli* [20] which demonstrated the leak-less properties of this system and persistence of state after removal of recombinase input. Here, we have constructed an artificial overlapped inversion switch by integrating two recombination systems (using the FimB protein of the *fim* system from *E. coli* [19] and the *hin* system from *Salmonella* [21]) to form an intercalated double inversion system that implements a heritable memory with finite state machine-like behavior with four states dependent on the sequence of invertase activity inputs. We expand on how this works below.

There exist only a few known examples of natural systems that utilize multiple overlapping DNA inversions for diverse gene expression. Some examples are the R64 plasmid shufflon [22], which uses inversion to select among different versions of PilV gene, and the Min system from the p15B plasmid [23], which can make 240 different isomeric forms of a phage protein. The natural example most parallel to our own is the nested inversion system of *Campylobacter fetus*, where a promoter is moved around via inversion near various S-layer protein genes for expression [24]. All of these systems are thought to be involved in extending the host range of their host pathogen. To date, our system is the first artificial system to incorporate two inversion systems into a single circuit for controlled DNA rearrangement. It is, also, perhaps, one of the first biological finite-state machine encoding more than two sequential states, along with the hin based inversion system used to solve a version of the burning pancake problem [25].

## Results and Discussion

### An informative idealization of invertase based Recombinatorics

Before delving into the experimental realization of our circuit we demonstrate an aspect of the possible power of this approach through a quick series of calculations of scalability of designs with invertase activities as input and resulting DNA sequence as the formal output or “state” of the system. In some cases it is possible to have a particular DNA configuration encode the expression of RNA and this could then be an output as well. How the actual physical realization of such circuits can affect the predictions of this idealization will be discussed briefly. In fact, it is critical to the experimental results of our circuit described below. But our goal in this work is not to create a biological computer but only to suggest the power of using DNA read/write as a “stateful” element in synthetic biological regulatory circuitry. Interestingly, an iGEM team from Davidson University has already considered using the possible computational power of a single invertible system as a means of solving a combinatorial problem [25]. As will become clear below, the nature of recombination operations in DNA result in combinatorial equations that describe both their configurations and operations. Thus, we call the theory of design with recombinases “Recombinatorics”.

A more complete theory would include, among other things, the wide variety of recombinase activities including excision, insertion and inversion of DNA segments into a target region of a replicon. Here we limit ourselves to invertases like those in our experimental implementation. For the purposes of our arguments here, we assume the following idealizations of invertase circuit dynamics, nearly all of which are violated in some way by our own circuit but also all of which are not beyond the ken of natural engineered inversion systems. First, we assume that there is a single copy DNA target for the recombinases whose activity serves as input to our system. Second, recombinases can only invert a target region once. That is, they are irreversible flippers. This also limits the possible computational power of the device quite a bit. Third, there is no interference among the recombinase inputs such that the ability of one recombinase to flip the region of DNA between its target pair of sites is unaffected by the presence of other recombinases or by the state of the surrounding DNA. Fourth, the length of DNA between a pair of inversion sites is such that the invertases can flip it. Fifth and finally, for simplicity, our input alphabet, which is the set of different invertases, is assumed to be presented as a sequence of single activities, each element of which is well separated in time and on long enough time-scale to effect a flip. No invertases are present more than once in a sequence. Thus, for N invertases there are N! possible ordered input sequences to our device.

**Figure 1:**
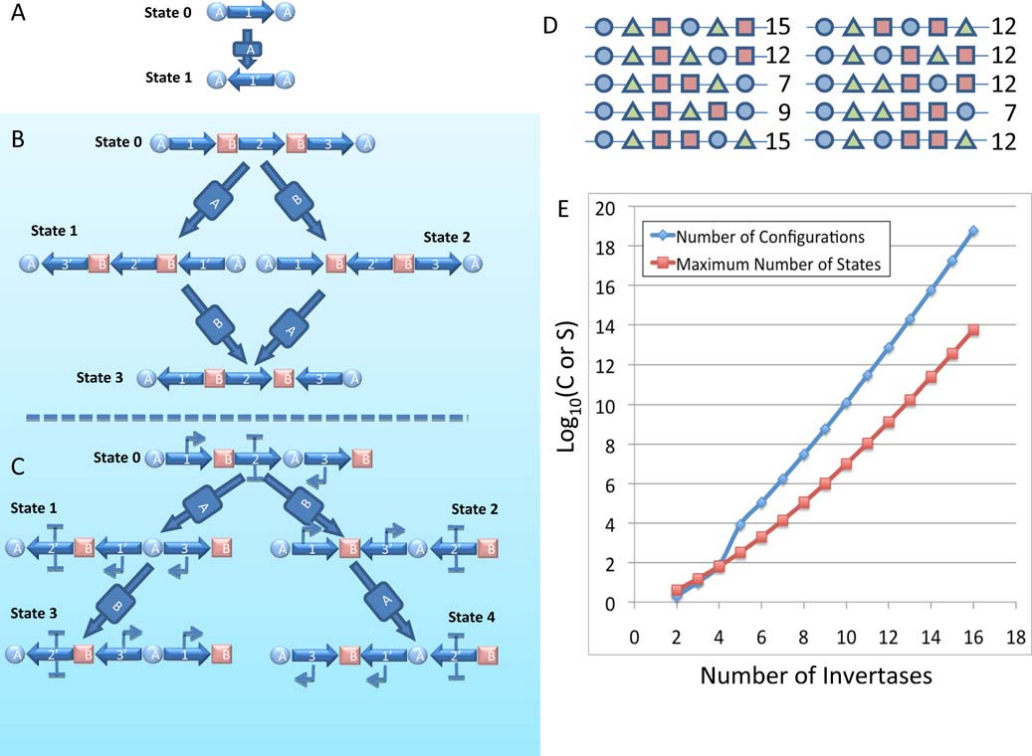
How invertase site configuration defines different “machine behaviors”. A) A single invertase can only flip one region ON or OFF. B and C) With two invertases there are two possible intercalated configurations of sites. In B, when one pair of sites is fully contained by another the resultant number of possible “states” of the system is only four, whereas in C, when the site pairs are staggered, five states are accessible and any input sequence results in a different output of the system. C also shows a configuration of promoters and terminators such that states 3 and 4 have promoters pointing outside the machine regions. This configuration is the one experimentally created. D) Possible configurations of three different pairs of recognition sites and the number of possible states. E) How both the number of such configurations (Blue curve) and the maximal number of states (Red curve) grows with number of pairs.

Our device is defined by an arrangement of the N pairs of inversion sites on DNA with no pair appearing more than once in the device. While there might be multiple devices encoded on a single duplex of DNA, we call a single (fully connected) device a set of pairs of sites for which every pair brackets a region of DNA that overlaps another region bracketed by at least one other pair of the device. Figure 1 shows possible arrangements of sites for devices accepting one (Figure 1A), two (Figure 1B&C) and three recombinase (Figure 1D) inputs. The number of such possible configurations increases rapidly with number of invertases. Assuming that configurations that are identical under shuffling of site identity are equivalent (that is, x-y-x-y is equivalent to y-x-y-x), an enumeration of all possible devices with n inputs a(n) suggests that with n = N-1, 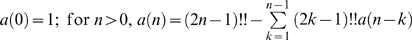, (Code for this and all enumerations is included in Supplementary Information S1. The inference of formulae from sequence was provided by the Online Encyclopedia of Integer Sequences.) By the time one has ten invertases there are more than 10^10^ possible arrangements of sites. The graph of possible configurations as a function of number of invertases, Figure 1E, shows the better than exponential increase in number of configurations as a function of N. If we assume we need at least 500 basepairs between sites for flipping to occur and each site is about 30 bp, a device with N inputs has a minimal size of around 30*2*N+500 basepairs (overlapping regions can decrease this slightly). For a device with 10 inputs then, apart from the DNA encoding the expression of the recombinases, 1.1 kilobases is all that is required to encode any of ten billion machines. This is the length of an average sized gene.

Each of these devices behaves differently under the N! possible inputs. Figure 1A–C shows the state transition graphs for all configurations of 1 and 2 invertase input devices. Each transition shows the transformation of one DNA state to another for each allowed sequence of inputs (see caption). Theoretically, for certain configurations, starting from an initial state of the device (state 0), it is possible that every possible history of input is recordable in the state of the DNA. That is, it is possible to determine which even partial sequence of inputs the device has seen by sequencing the DNA between its outermost sites. Simply counting the internal nodes of the state transition graphs like those in Figure 1C shows that the number of states (excluding state 0) for such devices is the number of permutations of non-empty subsets of {1,…,N} or 
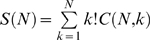
. A graph of this function is shown in Figure 1E. Devices accepting input from 10 invertases have maximal state-spaces of nearly 10^7^.This is a large space made accessible by addition of a region of DNA on the order of one gene in size (not counting the recombinases). Of course, not every configuration has this full state space. For example, figure 1C is the only configuration of two pairs of sites that has the full state. The other configuration (Figure 1B) has a cycle with the final states of the input sequences {A,B} and {B,A} being identical. Figure 1D shows all the configurations of three pairs of sites with the number of distinguishable states available to each shown to its right. Only two of the ten configurations show the full rank state-space. The others can “remember” only subsets of the input sequences. Nonetheless, as a system to remember which of a sequence of N inputs occurred, these types of devices could be immensely efficient in terms of size and operation.

These devices may be more than memory even under these restrictive assumptions. With proper placement of “active” elements such as promoters, terminators, and even genes within the device, the system can have active output at chosen states of the device. While a full treatment of how to place such elements within a device is beyond this paper, Figure 1C shows an example that we implement experimentally below. In this case, two promoters and a bi-directional terminator have each been placed in a separate DNA region. Under the operation of the two input recombinases, A and B, the regions containing these elements are rearranged. Only the two “end” states of the graph have an arrangement such that the promoters point away from the terminator and transcription may proceed outside the device boundaries. While the RNA produced might itself be an input to a downstream device, more classically genes may be placed to the left and right of the device and be differentially expressed based on whether the sequence A and then B occurred or the reverse. These genes could be recombinases that drive this device or other devices encoded into other regions of DNA or could be hooks into other more classical synthetic biological circuits. If we allow genes to be placed internal to the device, then all four states could have different output activity. How this ability scales to larger devices remains to be seen and affects how powerful a computer one could build with such a system if one were so motivated.

All this analysis concerns ideal systems however. If flipping is reversible, for example, our device in Figure 1C has an addition state with DNA configuration (3→2→1), that is reachable from the end states by action of either invertase A or B. While our fifth assumption above prevents us from reaching this state, reversibility means that the ordered input sequence {A,B} leads first to a probability of being in state 0 or state 1 when A is input, then to a probability of being in state 0, 1, 2 or 3 when B is input. What the ratio is of these probabilities is dependent on the kinetics of the forward and reverse flipping rates of the two invertases and the time they are allowed to be active. In this case, one only gets a probability of state 3 if A and then B and one only gets a probability of state 4 if B and then A so it is still possible, statistically, to determine the order in which inputs were seen from DNA sequence. Each member of a population of cells exposed to conditions that activated invertases A or B in a given order will hold one state of the DNA sequence, and a particular configuration can be read out by PCR or by a screenable output from the device. Violation of the other assumptions above leads to other interesting phenomena which may be either good or bad for specific applications. All the implications of such violations are beyond the scope of the paper other than the fact that in our experimental construct the effects end up being important.

### An Experimental implementation of a two invertase, full rank system: Basic design and construction of the device

To begin to understand the physical constraints on the building of invertases-based memory devices and switching elements, we chose to construct a device like that shown in Figure 1C. While we could have designed for states 1-4 of our device each to have a separate active output, to simplify our design and to make the measurements realizable we settled on a design with fluorescent proteins to the left and right of the device such that only states 3 and 4 would show output. However, as will be discussed below, the invertases we chose show reversible flipping and so the fifth state mentioned above also becomes accessible. If seen, this state would output both the left and right fluorescent proteins.

The *hin* and *fim* systems were chosen for constructing this new genetic switch for the following reasons, in addition to the ones that are obtained for any recombination circuit outlined above. 1) They have very specific recognition sites. These sites are well known and their DNA-protein interactions have been described. 2) They are independent of each other. Unlike inversion recombination systems that are from the same family, the *hin* and the *fim* systems are completely orthogonal with different mechanisms. 3) They are inducible. Only in the presence of the Hin and FimB proteins can the system invert. 4) Their mechanisms have been well studied. There is some twenty years of literature exploring the recombination mechanisms. 5) They are known to be flexible. The distance between the recombination sites can be varied greatly, from a few hundred base pairs to several kilo-bases. This allows the possibility of adding additional promoters or genes in between the recombination sites. 6) They have very low rates of excision, even when the inversion sites are arranged in direct repeats. Other inversion recombination systems (for example Cre/Lox) will excise the region between direct repeats.

Each inversion reaction was initiated by the expression of one of the recombinases, FimB or Hin. The gene encoding FimB was expressed from the arabinose-inducible *araBAD* promoter (P_BAD_) [26], and the gene encoding Hin was expressed from the aTc-inducible *tet* promoter (P_Tet_). Fim and Hin are expressed from a plasmid (pZB) containing the two promoters [27]. The recombinase genes were harbored on a separate plasmid from the switch to facilitate testing. Thus, the system has two inputs and two outputs. Although the two recombinases were expressed from inducible promoters, it would be possible to express them from a different input, for example environmental or metabolic sensors.

**Figure 2:**
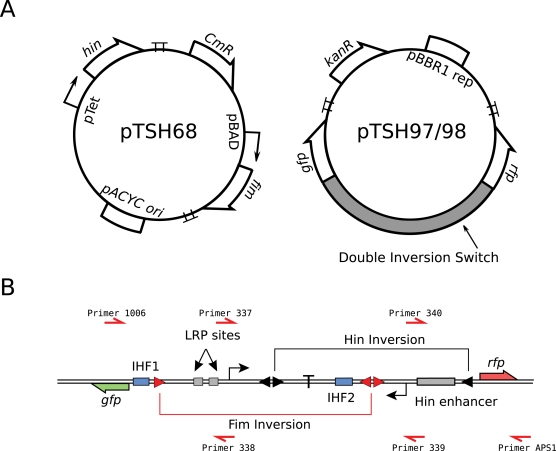
Plasmids used in the study. A) Plasmid maps for constructs containing the invertases and the memory switch. B) Detail of structure of the double inversion switch annotated with the primers used to diagnose state as described in the text. The Supplementary Information S1 contains the complete annotated sequence of the switch.

The target DNA regions were harbored on a separate plasmid. The switch region was synthesized *de-novo* and cloned into pPROBE-gfp. The red fluorescence gene was cloned in last. The fluorescent proteins GFP and RFP are placed to the left and right of the device respectively. The resulting plasmid is multicopy, meaning that there are many copies of our device in the cell such that the state of the device could possibly be different for each copy of the plasmid. Since Hin and FimB are reversible enzymes, each cell will harbor a mixture of states of the device as described below. The plasmids for the input and DNA response elements of the device are shown in Figure 2A.

### Recombinase optimization

The double inversion circuit requires two independently induced recombinases, FimB and Hin, to be expressed. In addition, they have to be repressed independently; that is, ideally, one can be induced while the other is repressed. Our switch was sensitive to leaky expression of Hin and FimB, so we required a very tightly controlled expression system. Currently, there are only a few tightly-controlled, regulatable expression systems available. One of the best is the P_BAD_ system, but this system cannot be used with a lac promoter, as they have significant crosstalk [28]. Thus, P_tet_ was used along with the P_BAD_ system on a single vector containing both (Fig. 2A), to express the recombinases independently. However, P_Tet_ did exhibit leaky expression and required remedy prior to use with our sensitive switch.

In addition to promoter optimization, translational efficiency was optimized. Seven different ribosome binding sites (RBSs) across a range of translational efficiencies were tested with the *hin* and *fimB* genes [16]. The optimization criteria were level of expression (does it cause inversion when expressed) and leakiness (does it cause inversion when not expressed). As our PCR based reporting method was very sensitive, the selection was biased against leakiness rather than towards high expression. In addition, degradation tags (LVA) or LacI binding sites were tested for their effects and possible efficacy at reducing background leaky expression (data not shown). The pTSH68 vector (containing *hin* with RBS sequence “AGGGACAGGATA” plus the LVA tag driven by P_Tet_ and *fimB* with RBS sequence “GAAGGTTCCTCA” driven by P_BAD_) was chosen as the recombinase expression vector.

### Target Device optimization

The two recombinase recognition sites in the middle flanking the terminator needed to be constructed in such a way that they form an inverted repeat even after a recombination event. As such, they had to be mirrored; that is, the binding site is repeated immediately adjacent, but in the opposite strand orientation, to maintain the inverted repeat orientation (Fig. 2B). The design for the switch is finalized by placing the necessary elements of both the *fim* inversion (2 IHF elements, LRP sites, the inverted repeats), and *hin* inversion (*hin* enhancer site, inverted repeats) along with generated sequences that separate the components (Fig. 2B). The enhancer elements are somewhat flexible in their location [29], [30], [31], so they were placed in similar places as the original switches. The entire switch, excluding *gfp* and *rfp*, is approximately 1 kbp. Fully annotated sequence of the switch is included in the Supplementary Information S1.

### Predicted and measured function of the switch

The heritable switch functions as shown in (Fig. 1C) and briefly outlined above. Figure 2B shows the actually constructed state 0. Hin plays the role of invertase B and FimB plays the role of invertase A. State 3 is only reached and GFP expressed when Hin is induced following FimB. State 4 is only reached, and RFP expressed when FimB is induced following Hin.

Because our inversion recombinases are reversible, two deviations from ideality are immediately expected. First given a long enough induction time, the invertases will switch back and forth between the two states its activity connects. Thus, the populations of these two DNA states in the plasmid population will reach some steady-state ratio. This is important to understand because when state 0 is induced to state 1, both state 0 and 1 are present in the population (either as mixed plasmids or cells). Ideally, if the reaction is allowed to go to steady state, there will be a 50/50 mixture of the initial and final states, as FimB and Hin are known to have the same forward and reverse rates. In our case, if FimB is expressed at state 0, we will end up with a 50/50 mixture of states 0 and 1. Subsequently, if FimB is removed, so that there are no further transitions between state 0 and 1, then Hin is expressed and allowed to equilibrate, ½ of state 1 will transition to state 3, and ½ of state 0 will transition to state 2. Thus, the overall population will have ¼ state 0, ¼ state 1, ¼ state 2, and ¼ state 3. A similar result ensues if we apply Hin and then FimB: only states 0, 1, 2, and 4 are populated. Second, because the invertases are reversible it is possible to apply them more than once in sequence. The sequences {Hin, FimB, Hin} and (FimB, Hin, FimB} both result in 1/8 of the population reaching the fifth state of DNA (3→2→1) mentioned above. Once state 5 is reached, the order information is lost, and the system no longer remembers which states it had been, but rather simply that it had seen both inputs.

Such mixture of states is not entirely desirable, but as there are no known unidirectional, re-settable switches available so we can restart our system, and having only one unidirectional switch (FimE) would unbalance our population distribution, the mixtures were accepted as a compromise for demonstrating our proof-of-principle circuit.

As should be clear from above, our device resembles an AND gate switch in electronic and logic circuitry. An AND gate only outputs when both inputs are present. This circuit, however, behaves differently than a regular AND gate. Instead of requiring that both inputs be present in order to have output, it remembers the input order—that is, it remembers that inputs *had* been present at some time in the past. The switch then outputs a different fluorescent protein, conditioned on the sequence of inputs it had observed. A temporal memory switch like this has many potential uses, such as in environmental sensors that can track two different conditions occurring one after another, or as *in vivo* biosensors investigating development or other temporally sensitive assays.

### Testing for the inversions

As many of the inversion states did not have fluorescent output, and because as discussed below flipping events were rarer than expected, the inversion state was assayed using “culture PCR” as described in the methods section. A culture PCR is similar to colony PCR, except the template is not a single colony, but part of the induced culture, likely containing a mixture of inverted genotypes. Six different primers in four permutations were used to probe for the presence or the absence of PCR products (Tables 1, 2 and Fig. 2B). The assay was chosen because, as the orientation of the DNA fragments change, different permutations of primers will amplify different length segments or generate no product at all. This method allowed us to detect very sensitively the presence or absence of a certain orientation of DNA, based on the presence or absence of PCR amplification product of different lengths. Culture PCR was much more accurate than detection by fluorescence, as first generation inversion (states 1 and 2) would show no fluorescence and, it turns out, production of the end states is excessively rare. The sensitive nature of PCR amplification allowed the detection of rare inversions. The inversion state was also verified by sequencing of the PCR product bands and by direct sequencing of the vector.

**Table 1:** Culture PCR primer sets and lengths of their PCR products. See Fig. 2B for their locations on the switch.

Primer Set	Description	Primers Used	PCR Product Length
E	State 0	337, 339	Short
F	States 2 and 3	337, 1006	State 2: Short, State 3: Long
G	States 1 and 4	339, APS1	State 1: Short, State 4: Long
H	State 5	338, 339	Short

**Table 2:** Sequences of primers used to interrogate the inversion products.

Primer Name	Sequence
337	cgagccacagaaacgttagctttacatatagcg
338	cgctatatgtaagctaacgtttctgtggctcg
339	cgcgacacgtggcgagtatatgatg
340	catcatatactcgccacgtgtcgcg
APS1	cgaattggggatcggaag
1006	caagaattgggacaactcc

**Figure 3:**
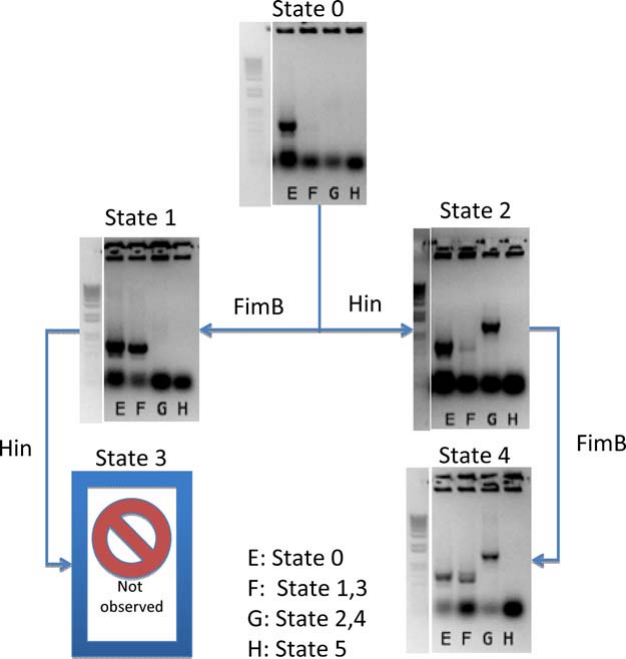
Culture PCR results for the response to the different input sequences. {FimB}, {HinB}, {FimB, HinB} and {HinB, FimB}. Each lane probes for the existence of a state or states, as shown in the legend. Because of the possible arrangements of DNA, lanes E and F could result in either short or long PCR products, indicating states {1, 3}, and {2, 4} respectively. See Figure 2 for relative location of the primers. No State 5 was observed when Hin and FimB were simultaneously expressed.

Figure 3 shows the results of our experiments. In these experiments cells were grown in LB at 37°C and exposed to either arabinose or tetracycline. The *hin* mirrored pair was replaced with *hixC*, a variant inversion sequence which allows inversion in both directions without impediment [13], as the *hin* mirrored pair behaved unpredictably due to its native mirrored-pair like structure (data not shown). *HixC*, however, does have a side effect of resulting in a higher rate of self-excision of the DNA when in a directly repeated orientation.

The *fim* inversions performed as expected, except with a much lower inversion efficiency than *hin*. By culture PCR, we determined that some fraction of the cells were inverting, resulting in strong PCR product bands. However, of the 50 isolates sequenced, none of them had the inverted *fim* orientation, even though sequencing of the PCR product showed clean *fim* inversion. It would be possible to screen more perhaps by doing a serial dilution and a binary tree search. It appears that the intercalated switch construct has low *fim* inversion efficiency, with an unknown rate.

In all inversions, inducing the culture at low OD rather than stationary phase resulted in a greater number of inversions. For *hin* inversion, this is consistent with the fact that Fis production is eliminated during stationary phase [32]. However, it does not explain the increase in *fim* inversion. Perhaps IHF or LRP is regulated in a similar fashion as Fis, or there is an unknown protein involved in *fim* inversion.

The rarity of *fim* inversion had consequences for the second generation (states 3 and 4) inversions. State 4, the *hin* then *fim* inversion, although rare, was observed by PCR product sequencing, as a large fraction of *hin* inversion occurred, and a small fraction of *fim* inversions then occurred. However, state 3, the *fim* then *hin* inversion, was never observed. This is perhaps the result of only having a small fraction of *fim* inversion occurring, and among the smaller pool of inverted *fims*, the occurrence of *hin* inversion was very small. It may also be that the *hin* inversion efficiency was lower than normal due to the DNA rearrangement via the *fim* inversion, further suppressing the occurrence of state 3 plasmids.

Many of the problems encountered in creating a functional double inversion switch stemmed from two principal causes. First, the necessity for the mirrored pair introduced a very long hairpin structure that is both difficult to construct and maintain within *E. coli*. Perhaps strains more tolerant of such structures would be useful. Also, such hairpins have made sequencing very challenging: constructs containing the mirrors had to be sequenced piecemeal, as the sequencing reaction would stop at the very edges of the hairpin. Additionally, the hairpins acted as terminators of unknown strength for the internal constitutive promoters, although this had a positive effect on our switch design.

The second problem is our lack of understanding about the exact structure of protein-DNA complex that forms when FimB binds to the inversion sites. The assumption of non-interference between recombinases was clearly violated. Perhaps a better understanding of the structure of FimB, including its reaction with the enhancer and DNA bending proteins, would reveal insight into why the switch did not perform as expected. As a whole, however, the switch did demonstrate history dependent configurations with states 0, 1, 2, and 4 visited as expected although not with the frequency desired.

### Conclusions

A heritable inversion switch was designed and constructed using both the *fim* and the *hin* inversion recombination systems. The design allowed for encoding of state information to the DNA, which would be inherited generation after generation. The design also displayed finite state machine-like behavior, as the reporter would transition to and from different states, recording path traversal as it went along.

The switch as constructed was able to transition into three out of the five end states (excluding initial state 0). Thus, we could detect the following sequences: Hin alone, FimB alone, or Hin followed by FimB. It was unable to transition to the other two states, FimB followed by Hin and (Hin:FimB:Hin, FimB:Hin:FimB), probably due to poor transition rates of the *fim* inversion. The mechanism for the poor transition rate was not determined.

In order to construct a fully functioning heritable switch as envisioned in this paper, robust inversion systems are a necessity. The *hin* system, using *hixC*, is quite robust to introduction of exogenous sequences, including strong hairpins and other recombination sites. The *fim* system, however, seems to be less robust, perhaps because it is more sensitive to the positioning of the IHF and LRP binding sites. Or it is possible that there are yet to be discovered key mechanisms not considered in the design. A greater understanding of the *fim* inversion system is necessary for the development a robust system. In this study there was not an opportunity to construct a variety of *fim* inversion systems with many different arrangements of IHF, LRP, mirrored pairs, terminators, in different permutations to concretely discover the exact cause of the inversion repression. With the existing synthesis technology, a study that would rigorously test the necessary elements for *fim* inversion would have been very costly and time consuming. But perhaps with advanced DNA synthesis and assembly technologies, such tests may be feasible in the near future. Because of the apparent power and theoretical efficacy of these devices for encoding states it seems a useful program on which to embark.

Despite its limitations, this work creates a proof of principle mechanism for production of finite-state DNA read/write systems and has uncovered key challenges that might not have been clear without the construction. With the large variety of enzymes that edit DNA, successful harnessing of DNA recombination systems could lead to powerful applications in biological control and sensing.

## Materials and Methods

### Construction

All standard molecular techniques were performed using established protocols [33]. The double inversion switch was synthesized *de novo* in multiple steps. First, a simplified version of the switch, which had the *fim* and *hin* mirrored pairs and the middle terminator replaced by restriction sites, was synthesized using the protocol of Rouillard and colleagues [34]. The product was cloned into the pPROBE vector [35], which harbors *gfp* encoding the green fluorescent protein along with flanking terminators, resulting in pTSH49. Once the sequence was verified, the mirrored pairs and the terminator were cloned in via double strand oligo ligation. Four oligos were synthesized, which when annealed together left a sticky end corresponding to the desired restriction site. The oligos were diluted to 20 μl, and complementary sets were annealed together by mixing, heating to 95°C for 10 minutes, then cooling to room temp gradually. The annealed oligos were kinased, and finally mixed with the vector in a ligation reaction, producing pTSH117, with 10 bp within the *fim* and *hin* mirrored inverted repeats, and pTSH118, with 30 bp within the *fim* mirrored inverted repeats and 10 bp within the *hin* mirrored repeat.

The strong hairpins formed by the mirrored pairs prevented sequencing through them, so their successful insertion was verified by checking the insert length via PCR and by the presence of an un-sequenceable hairpin. A construct containing *hixC* in place of the *hix* mirrored pairs was also made (pTSH89, 10-bp gap *fim* mirrored repeat, pTSH90, 30-bp gap *fim* mirrored repeat). The completed switch, which includes *hixC,* the mirrored *fim* repeats, the terminator, *gfp* and *rfp*, were named pTSH97 (10 bp gap *fim* mirrored pair) and pTSH98 (30 bp gap *fim* mirrored pair), respectively.

### Testing

The testing of the double inversion switch was performed *in vivo*, in *E. coli* DH10B. The vector containing the switch, along with any reporter fluorescent genes, was co-transformed with another vector containing the recombinases *fimB* and *hin*. The expression of FimB and Hin was performed through induced expression via P_BAD_ (*fimB*) and P_Tet_ (*hin*). The ribosome binding sites (RBS) of the genes were mutated to adjust the expression level. This was done via addition of overhanging sequences during PCR.

Induction experiments were performed as follows. The strain was grown at 30°C overnight in LB medium containing kanamycin (50 μg/ml), chloramphenicol (30 μg/ml), and dextrose (5% w/v). Dextrose was added to prevent spurious P_BAD_ expression. The overnight culture (50 μl) was inoculated into 2 ml LB medium containing kanamycin and chloramphenicol and incubated at 37°C with shaking. After reaching an OD_600_ of 0.2 to 0.5, the inducer (10 mM arabinose for *fimB* induction and/or 100 nM anhydrous tetracycline (aTc) for *hin*) was added. The culture was incubated at 37°C with shaking for 6 hours or overnight. Control cultures were inoculated into non-inducing medium. To test for states 3 and 4, the cultures in states 1 and 2 were inoculated into fresh medium (50 μl of culture into 2 ml of LB) without an inducer, grown overnight, and induced with the complement inducers.

After the prescribed induction time, a 1 ml sample of the culture was centrifuged (15,000×g, 1 minute), and the pelleted culture was used as the template for four PCR reactions (“Culture PCR”). Four primer sets were used: 337 and 339 (which checks for state 0); 337 and 1006 (checks for states 2 and 3); 339 and APS1 (checks for states 1 and 4); and 338 and 340 (checks for state 5). They are labeled as primer sets E, F, G and H, respectively (Tables 1 and 2). The culture PCR was performed as follows. A PCR master mix (containing 2.5 μl 10×Taq PCR buffer, 0.2 μl 25 mM dNTP's, 0.2 μl taq polymerase, 0.5 μl 20 uM primers in 20.6 μl nuclease free water) was mixed with 0.5 μl pelleted cells. The PCR cycling parameters were 95°C for 2 minutes, 35 cycles of (95°C for 30 seconds, 55°C for 30 seconds, 72°C for 90 seconds), then 72°C for 2 minutes, finally, hold at 4°C.

The PCR products were run on 1% agarose gels, and visualized by ethidium bromide stain under UV light. Sometimes the bands were cut and gel extracted for sequencing. The vector itself also was sequenced for state identification. Because different copies of a multi-copy vector might harbor different inversion switch states, which would make sequencing impossible, plasmids were mini-prepped after the induction and transformed into *E. coli* to isolate individual plasmids for sequencing.

## Acknowledgments

APA would like to thank Tom Knight of M.I.T. for pointing out the amazing Online Encyclopedia of Integer Sequences which allowed us to be lazy about proofs.

## Supporting Information

Supplementary Information S1 (0.07 MB DOC)
