# Supplementary material for: Design and Construction of a Double Inversion Recombination Switch for Heritable Sequential Genetic Memory
Source: PLoS One. 2008 Jul 30;3(7):e2815. doi: 10.1371/journal.pone.0002815 (PMC2481393; doi:10.1371/journal.pone.0002815)
Supplement: Supplementary Information S1 — (0.07 MB DOC) [file pone.0002815.s001.doc]

# Supplementary information

# Design and Construction of a Double Inversion Recombination Switch for Heritable Sequential Genetic Memory

### Timothy S. Ham^1^, Sung K. Lee^2^, Jay D. Keasling^123^, Adam P. Arkin^12*^

## A. Annotated Sequence for the Constructs Schematically Represented in Figure 2, excluding gfp and rfp. Same colors are used as in fig. 2.

>sequence checked #98

IHF1 Fim IRL

gatttaactt attgataata aagttaaaaa aacaaataaa tacaagacaa ttggggccat

LRP

tttgactcat agaggaaagc atcgcggaca aactttttta gtttatttgt tggcttaata

LRP

ttctattgtt atctttattt atagatgttt atattgcatg aggtggtttt tggagagaag

primer (337, 338-rc)

aatgaggaag atgcgtcgag ccacagaaac gttagcttta catatagcgc cacaccagag

(372) promoter

ctaagcgctg gctattaaaa cggatcacag ctatgcttga gaaaaatacg taacttattt

hixC

atgatagatt taagggcttg cctcagcaac tagagtcaag aggtttatcc taaaccatggt

ttaggataaa tcggtgtaac ccggggctgga cgttaagagg cgacgatacc

Primer (373)

gaataatgtc atcggtgacg ctgtacccct aaggggtacc agtggcctag tatcctctat

gaccacgtga tcccgatgtt ccagagagcg tgcatcttta tcgaggtgat gtgaaattaa

IHF2

tttacaatag aaataattta catatcaaac agttagatgc tttttgtcgt ttgagctc

Fim IRR10

Tttaatacag tttggcccca aatgtttcat cttttggggg aaaactgtgc agtgttggcag tcaaacCTGG AGGTCTTCAa acactgcaca gttttccccc aaaagatgaa acatttggggc caactgtatt aaagagctctt

promoter

ctgtcgctgt ggaaataagg aatctgaaaa attagacgta tcataccacg tcccccctga

gatgtcaagc atagtgcgga tacagagagt gggcgaacgg atacactacc ccatactggg

primer (340(rc for the promoter), 339)

tgggccagat gcatcatata ctcgccacgt gtcgcgtgaa ccgccacgca ctccggtaag

hin enhancer region

gggggtacat at tacatact ttaacgctcg tttcaggccg gggcggtttg caatcttgcc

actgatacgg tcctcaaaaa tgcggtcaca atttgcacta gtaagcgcat tacgctgtaa

atcgatattt tggtcaattg ttgacacccg aatataccca atagtagcca tgattttctc

hixC

ctttacatca gataaggaag aattttagtc gcttttctca tggaggattg ctttatccta aaccatggtttaggataa tctaga

***B. Fim inverted mirror pair variants. Yellow represents complementary sequences, capitals represents the gap between the complements. Red text represents the recognition site for the Fim recombinase.***

>fim IRR 10

Gagctcttta atacagtttg gccccaaatg tttcatcttt tgggggaaaa ctgtgcagtg

Ttggcagtca aacCTGGAGG TCTTCAaaca ctgcacagtt ttcccccaaa agatgaaaca tttggggcca aactgtatta aagag

>fim IRR 30

Gagctcttta atacagtttg gccccaaatg tttcatcttt tgggggaaaa ctgtgcagtg

Ttggcagtca aacCTGGAGG TCTTAAACAC CTGCCAGCTa acactgcaca gttttccccc aaaagatgaa acatttgggg ccaaactgta ttaaagagct c

## C. Script for Enumerating Configurations and States for Invertase Machines.

#!/usr/bin/perl -w

#

# MakeInverterConfigs.pl

#

# A program to enumerate number of site configurations and number of

# states per configuration in

# intercalated inversion switch designs.

#

# Author: Adam Arkin

# Date: 01/27/2008

#

#

use strict;

use warnings;

# Output for number of configurations. Visualize using GraphViz.

my $dotfile="ConfigurationGraph.dot";

my $configfile="ConfigurationFile.txt";

# Start from simple one site case. This is just pairs of alphabetic

# sites A, A' separated by the

# number '1' which we will use later.

my $initconfig="{A1A'1}";

my $configtree={};

$configtree->{"self"}=$initconfig;

$configtree->{"children"}=[];

# This is the key function that adds sites to a given config.

# It is recursive.

# The depth of the tree is hard-coded in the algorithm below.

AddObject($configtree,1,2);

# print to the output files. the dot file is for graphviz

my $nstr="";

my $gstr="";

$nstr .= "/*\n";

$gstr .="digraph configs {\n";

my $level=1;

my $childcnt={};

PrintConfigTree($configtree,\$nstr,\$gstr,$level,$childcnt);

foreach my $key (keys %$childcnt)

{

$nstr .= $key."\t".$childcnt->{$key}."\n";

}

$nstr .= "*/\n";

print $nstr;

print $gstr."}\n";

# this algorithm-- when used-- finds the number of unique states

# accessible by inverting between pairs of sites.

#FindStates("{A1B1A'1C1B'1D1C'1D'1}");

exit;

sub FindStates

{

my $config=shift;

$config =~ s/{|}//g;

my @parts=split('1',$config);

my $actions=[];

for( my $i=0; $i<=$#parts; $i++)

{

my $j;

if(length($parts[$i])!=1) {next;}

for( $j=$i+1; $j<=$#parts; $j++)

{

if( $parts[$j] =~ m/$parts[$i]/)

{

$j--;

last;

}

}

# print STDERR "$parts[$i]: $i - $j\n";

push(@$actions,"$i,$j");

}

my $N= $#$actions;

my @ops=(0..$N);

my $nreg= $#parts-1;

if($#ops== 0) {return;}

my $num_permutations = factorial(scalar @ops);

my $statetree={};

$statetree->{"Node"}=join("",0..$nreg);

$statetree->{"Children"}=[];

my $states={};

for (my $i=0; $i < $num_permutations; $i++) {

my @permutation = @ops[n2perm($i, $#ops)];

my $currentpermutation = join("",@permutation);

my @state=(0..$nreg);

foreach my $input (@permutation)

{

my @action=split(',',$$actions[$input]);

my @region=@state[$action[0]..$action[1]];

@region=reverse(@region);

for(my $j=0; $j<=$#region; $j++)

{

my $seg=$region[$j];

if($seg =~ /!/)

{

$seg =~ s/!//g;

} else {

$seg=$seg."!";

}

$region[$j]=$seg;

}

splice(@state,$action[0],$#region+1,(@region));

$states->{join("",@state)}++;

}

}

print $num_permutations*($#ops+1)."\t".keys(%$states)."\tstates $config\n";

}

sub PrintConfigTree

{

my $tree=shift;

my $nstr=shift;

my $gstr=shift;

my $level=shift;

my $ccnt=shift;

# Print Numbers #

my $ctree=$tree;

my $self=$ctree->{"self"};

FindStates($self);

my $children=$ctree->{"children"};

$ccnt->{$level} += scalar(@$children);

my $tch=0;

$level++;

foreach my $child (@$children)

{

my $cid=$child->{"self"};

my $cca=$child->{"children"};

$$gstr .= "\"$self\"\t->\t\"$cid\";\n";

$tch += @$cca;

if(scalar(@$cca)>0)

{

PrintConfigTree($child,$nstr,$gstr,$level,$ccnt);

} else { FindStates($cid);}

}

# $$nstr .= "$level\t".$tch."\n";

}

sub AddObject

{

my $ctree = shift;

my $N=shift;

my $L=shift;

my $target= chr(ord('A')+$N-1);

my $insert= chr(ord('A')+$N);

my $self=$ctree->{"self"};

my $children=$ctree->{"children"};

# Now we can insert anwhere to the left of the N-1 right bracket and before the leftmost bracket

my $cnt=0;

my @parts=split(/${target}1/,$self);

# print "--------- $N/$L -------\n))) @parts\n\n";

my $left=$parts[0]."${target}1";

my @rightparts=split(//,$parts[1]);

my $i=0;

my $c=0;

do

{

my $brack=$left."${insert}1".join('',@rightparts);

# print "--> $brack\n";

my @variants=PlaceRightBracket($brack,$insert);

foreach my $cself (@variants)

{

my $childp={};

$childp->{"self"}=$cself;

$childp->{"children"}=[];

push(@$children,$childp);

}

# print ":::: @variants\n";

# print "==> @rightparts\n";

if($rightparts[3] eq "}") {$i=10;}

if($i!=10)

{

$left .= "$rightparts[0]$rightparts[1]";

shift @rightparts; shift @rightparts;

if($rightparts[0] eq "1")

{

$left .= $rightparts[0];

shift @rightparts;

}

}

} while($i<4);

if($N<$L)

{

foreach my $childp (@$children)

{

AddObject($childp, $N+1,$L);

}

}

}

sub PlaceRightBracket

{

my $config=shift;

my $insert=shift;

my @parts=split(/$insert/,$config);

my $left=$parts[0]."${insert}";

my @targets=split(/1/,$parts[1]);

my @variants;

for(my $i=0; $i<$#targets; $i++)

{

my $ll= $left.join("1",@targets[0..$i]);

my $right = "1${insert}'1".join("1",@targets[$i+1..$#targets]);

push(@variants,"$ll$right");

}

return(@variants);

}

##############

#

#mjd_permute derived from PERL Cookbook

#

##############

# Utility function: factorial with memoizing

BEGIN {

my @fact = (1);

sub factorial($) {

my $n = shift;

return $fact[$n] if defined $fact[$n];

$fact[$n] = $n * factorial($n - 1);

}

}

# n2pat($N, $len) : produce the $N-th pattern of length $len

sub n2pat {

my $i = 1;

my $N = shift;

my $len = shift;

my @pat;

while ($i <= $len + 1) { # Should really be just while ($N) { ...

push @pat, $N % $i;

$N = int($N/$i);

$i++;

}

return @pat;

}

# pat2perm(@pat) : turn pattern returned by n2pat() into

# permutation of integers. XXX: splice is already O(N)

sub pat2perm {

my @pat = @_;

my @source = (0 .. $#pat);

my @perm;

push @perm, splice(@source, (pop @pat), 1) while @pat;

return @perm;

}

# n2perm($N, $len) : generate the Nth permutation of $len objects

sub n2perm {

pat2perm(n2pat(@_));

}
